# From Concept to Claim: An Integrated Lifecycle Roadmap (ILR) for Patient-Centric Endpoints in Clinical Trials and Real-World Evidence

**DOI:** 10.3390/healthcare14101299

**Published:** 2026-05-11

**Authors:** Moustafa Kardjadj

**Affiliations:** CRO Division, dicentra, Toronto, ON M4W 3E2, Canada; moustafa@dicentra.com or drkardjadj@live.fr

**Keywords:** patient-centric endpoints, estimands, digital health technologies, clinical trials, real-world evidence, computable phenotypes, clinical outcome assessments

## Abstract

**Background**: Patient-centric endpoints increasingly influence clinical development, regulatory evaluation, and health technology assessment, yet the relevant methodological guidance remains distributed across separate domains. Estimands, digital health technologies, and real-world evidence are often addressed independently, making it difficult for sponsors to apply them within a single endpoint development strategy. This review synthesizes existing guidance and framework literature to organize these elements into a coherent lifecycle perspective for endpoint development. **Methods**: This semi-structured narrative review used a structured search of PubMed, Embase, and regulatory databases (FDA and EMA) for records published from January 2009 through December 2025. We synthesized authoritative guidance and peer-reviewed validation frameworks, including V3 and STaRT-RWE, and assessed landmark precedents (SV95C, ADAPTABLE, and RW-Response) selected a priori using pre-specified criteria for regulatory relevance, technical reporting, and first-of-kind pathway relevance. **Results**: The literature supports four recurring requirements for patient-centric endpoint development: early concept elicitation to establish meaningful aspects of health, explicit estimand specification to address intercurrent events, staged validation of digital measures under V3 principles, and formal validation of computable phenotypes for real-world evidence use. Across the reviewed sources, a recurring gap was the lack of an integrated framework linking these components across the evidence lifecycle. To address this, we organize existing guidance into an Integrated Lifecycle Roadmap that connects concept definition, measurement validation, causal inference, and real-world transportability. **Conclusions**: Patient-centric endpoints are most robust when they are developed as part of a continuous evidence lifecycle rather than as isolated study variables. The proposed roadmap does not introduce a new methodology, but it provides a practical structure for aligning existing regulatory and methodological frameworks to support scientifically rigorous and patient-relevant endpoint development.

## 1. Introduction

In the hierarchy of clinical evidence, endpoints serve as the decisive evidentiary anchor upon which regulatory approvals, reimbursement decisions, and clinical practice guidelines are built. An endpoint is not merely a data point; it is a meticulously defined variable that quantifies a therapy’s impact on a patient’s life. Historically, the drug and medical device development paradigm was predominantly clinic-centric, prioritizing objective laboratory values, imaging markers, or composite clinical events, often relying on surrogate measures that, while statistically efficient, sometimes failed to correlate with actual clinical benefit [1]. This reliance on surrogate or clinician-derived measures has been repeatedly questioned in contexts where statistical significance does not translate into patient-relevant benefit, highlighting a structural misalignment between measurement and meaning.

The evolution toward patient-centricity has progressed through distinct eras. The initial shift in the 1990s and early 2000s established the foundational importance of patient-reported outcome (PRO) measures to capture functional autonomy and quality of life [2,3]. Recent systematic evidence highlights that while these tools bridged the gap between clinic-based assessments and patient experience, they often remained siloed from the broader statistical and real-world evidence (RWE) infrastructure [4,5]. Consequently, although patient-centricity became conceptually embedded in endpoint development, its operationalization remained fragmented, with limited integration into trial estimands, digital measurement systems, or post-approval evidence generation.

Over the past decade, this landscape has undergone a fundamental transformation driven by converging regulatory and technological developments. The introduction of the ICH E9(R1) addendum reframed treatment effects through the formalization of estimands, requiring explicit alignment between the clinical question, the population, the variable, and the handling of intercurrent events (ICEs) [6,7]. This shift has elevated endpoint definition from a descriptive exercise to a formal causal framework, directly linking measurement to inferential validity. In parallel, the rapid expansion of digital health technologies (DHTs) has enabled continuous, real-time capture of patient data outside traditional clinical settings. However, this innovation has introduced new challenges related to verification, analytical validation, and clinical validation, as emphasized by emerging V3 frameworks [8,9,10]. At the same time, the growing regulatory emphasis on real-world evidence (RWE) has extended the evidentiary burden beyond controlled trials, requiring that endpoints demonstrate robustness, transportability, and interpretability in routine clinical practice [11,12,13,14].

Despite these advances, the field remains characterized by a critical lack of integration. Estimand frameworks, digital measurement validation, and RWE methodologies are often developed and applied in parallel rather than in coordination. This disconnect creates a methodological gap in which endpoints may be valid within one domain (e.g., psychometric validity or regulatory acceptance) but fail to achieve coherence across the full evidence lifecycle. For example, a digitally derived endpoint may be technically validated yet poorly aligned with the estimand, or a trial endpoint may be statistically well defined but not reproducible in real-world settings.

Current literature has largely addressed these domains in isolation, with limited efforts to provide a unified, lifecycle-oriented framework that connects patient-centered measurement, causal inference, and real-world applicability. This gap is particularly relevant in the context of increasing regulatory scrutiny on endpoint justification, as well as the growing need to align clinical trial outcomes with health technology assessment (HTA) and payer expectations.

In response, this review proposes an Integrated Lifecycle Roadmap (ILR) that brings together estimand specification, measurement validation (including V3 principles), and real-world evidence generation into a coherent framework for endpoint development. The objective is not to claim a new methodological paradigm, but to synthesize and organize existing guidance into a practical, regulatory-informed model that supports the development of endpoints that are scientifically rigorous, technically validated, and meaningfully anchored in patient experience across the full evidence continuum.

## 2. Methods

### 2.1. Review Type: Narrative (State-of-the-Art) Review

This paper is a narrative (state-of-the-art) review. The rationale for this approach is to provide a high-level conceptual synthesis of rapidly evolving regulatory, methodological, and technological paradigms, specifically the integration of the ICH E9(R1) estimand framework with digital health technologies (DHTs) and real-world evidence (RWE). Given the multidisciplinary nature of this topic, which spans clinical trial design, psychometrics, data science, and regulatory policy, a narrative format was selected to support the development of an Integrated Lifecycle Roadmap (ILR) rather than an exhaustive catalog of all empirical studies. We acknowledge that this is not a PRISMA systematic review; therefore, the synthesis is inherently subject to expert judgment. To improve transparency, the search strategy, screening process, and case-selection criteria were pre-specified and documented.

### 2.2. Literature Selection and Search Strategy

While this is not a systematic review, a structured and transparent search approach was employed to identify foundational frameworks and landmark precedents. Searches were conducted for records published between 1 January 2009 and 31 December 2025. The following database-specific search strings were applied.

- **PubMed:** ((“patient-centric endpoint” OR “patient-centered endpoint” OR “patient reported outcome*” OR “patient-reported outcome*” OR “clinical outcome assessment*” OR estimand* OR “intercurrent event*” OR “digital health technolog*” OR BioMeT* OR “real-world evidence” OR “real world evidence” OR “computable phenotype*” OR psychometric* OR “content validity”) AND (“2009/01/01”[Date-Publication]: “2025/12/31”[Date—Publication]))**
- **Embase:** (‘patient-centric endpoint’:ti,ab,kw OR ‘patient-centered endpoint’: ti,ab,kw OR ‘patient-reported outcome*’: ti,ab,kw OR ‘clinical outcome assessment*’: ti,ab,kw OR estimand*: ti,ab,kw OR ‘intercurrent event*’: ti,ab,kw OR ‘digital health technolog*’: ti,ab,kw OR biomet*:ti,ab,kw OR ‘real-world evidence’: ti,ab,kw OR ‘real world evidence’: ti,ab,kw OR ‘computable phenotype*’: ti,ab,kw OR psychometric*:ti,ab,kw OR ‘content validity’:ti,ab,kw) AND [2009-2025]/py**
- **FDA.gov:** (“patient-reported outcome” OR “clinical outcome assessment” OR estimand OR “intercurrent event” OR “digital health technology” OR BioMeT OR “real-world evidence” OR “computable phenotype” OR “content validity”)
- **EMA.europa.eu:** (“patient-reported outcome” OR “clinical outcome assessment” OR estimand OR “intercurrent event” OR “digital health technology” OR BioMeT OR “real-world evidence” OR “computable phenotype” OR “content validity”)

The review prioritized authoritative sources, including primary regulatory guidance, qualification opinions, and consensus methodological frameworks. Where multiple versions of a guidance existed, only the most recent document within the search window was retained, unless an earlier document remained the canonical foundational source for a concept. For example, newer FDA and EMA documents on DHTs and RWE were retained preferentially over superseded versions, while foundational guidance documents were retained when they remained the authoritative source for the topic.

Inclusion criteria were: (i) primary regulatory guidance, qualification opinions, or technical standards; (ii) peer-reviewed methodological papers and consensus frameworks; and (iii) empirical reports with direct relevance to measurement validity and causal inference. Exclusion criteria were duplicate records, documents not directly relevant to the review question, purely opinion-based pieces, and papers lacking sufficient methodological detail to inform the ILR.

Case studies were selected as landmark precedents using pre-specified criteria: regulatory qualification or acceptance, availability of a public technical dossier, frequency of citation in regulatory or methodological discussions, and first-of-kind relevance for a digital or RWE-derived endpoint pathway. On this basis, SV95C, ADAPTABLE, and RW-Response were retained as exemplar cases.

### 2.3. Data Synthesis and Framework Development

The extracted evidence was synthesized qualitatively into a reinforcing evidence stream linking estimand specification, measurement validation, and RWE phenotype development. This synthesis informed the proposed Integrated Lifecycle Roadmap (ILR) and the operational checklists presented in the review. Because the study is narrative in design, the synthesis is interpretive rather than meta-analytic and should be understood as a structured conceptual framework, not as a quantitative evidence map.

### 2.4. Search Yield and Retained Evidence

The structured search strategy identified a focused body of literature and regulatory materials that were retained for synthesis in this review. The final evidence base was intentionally concentrated on authoritative guidance documents, consensus methodological frameworks, and landmark empirical precedents that directly informed the Integrated Lifecycle Roadmap (ILR).

At the guidance level, the retained corpus included foundational and/or most recent documents addressing estimands, patient-reported outcomes, real-world evidence, and digital health technologies, including ICH E9(R1) and its training materials [4,13], FDA guidance on patient-reported outcomes and digital health technologies [8,15,16], FDA’s real-world evidence framework [6], EMA registry-based study guidance and RWE guidance [17,18], the CDER COA Compendium [9], and the DARWIN-EU report [19].

At the methodological framework level, the retained literature included key works on PRO development and content validity [10,11,20,21,22,23], estimand implementation and intercurrent event handling [12,24,25], V3 and simplified V3 validation for biometric monitoring technologies [5,15,26], real-world evidence study planning and reporting [7,27,28,29], and data model harmonization and phenotype validation [30,31].

At the empirical precedent level, the review retained landmark examples illustrating the practical application of these frameworks, including SV95C [14], ADAPTABLE [32], and RW-Response [33]. These studies were selected because they exemplify first-of-kind or highly influential pathways for digital or real-world evidence-derived endpoints.

Overall, the retained evidence base was sufficient to support the conceptual synthesis presented in this review, while remaining deliberately selective in order to prioritize high-authority sources most relevant to endpoint science, measurement validity, and inferential alignment.

## 3. Conceptual Foundations of Clinical Endpoints

A patient-centric endpoint strategy is built upon three mutually reinforcing pillars: conceptual relevance (does it matter?), measurement reliability (is it accurate?), and inferential alignment (does it answer the clinical question?).

### 3.1. Defining the Concept of Interest (COI)

The “Concept of Interest” is the specific aspect of a patient’s health, function, or feeling that a study aims to quantify. Identifying the COI requires a bottom-up approach, typically through Qualitative Concept Elicitation (CE). In this process, patients are interviewed to identify the meaningful aspects of health (MAH) most affected by their condition [8,11].

For example, in heart failure, while a clinician might prioritize “ejection fraction” (a surrogate), patients often prioritize the ability to “walk to the mailbox without breathlessness” (a functional concept). Regulators now expect evidence showing that the chosen endpoint maps directly to these MAH. This is the bedrock of content validity, ensuring the instrument captures the essence of the patient experience without irrelevant noise [2,34].

### 3.2. A Strategic Taxonomy of Endpoints

Choosing the right measurement modality is a strategic decision that balances patient burden against regulatory requirements. We expand our taxonomy to highlight the trade-offs inherent in the ILR (Table 1).

### 3.3. Measurement Science: Beyond “Fit-for-Purpose”

To be accepted as a primary endpoint, an instrument must meet the COSMIN criteria [35], which assess reliability, validity, and responsiveness. Perhaps the most critical for regulatory acceptance is interpretability, defining the Minimal Clinically Important Difference (MCID). This is typically achieved through anchor-based methods, linking a score change to a patient’s Global Impression of Change [21].

**Figure 1:**
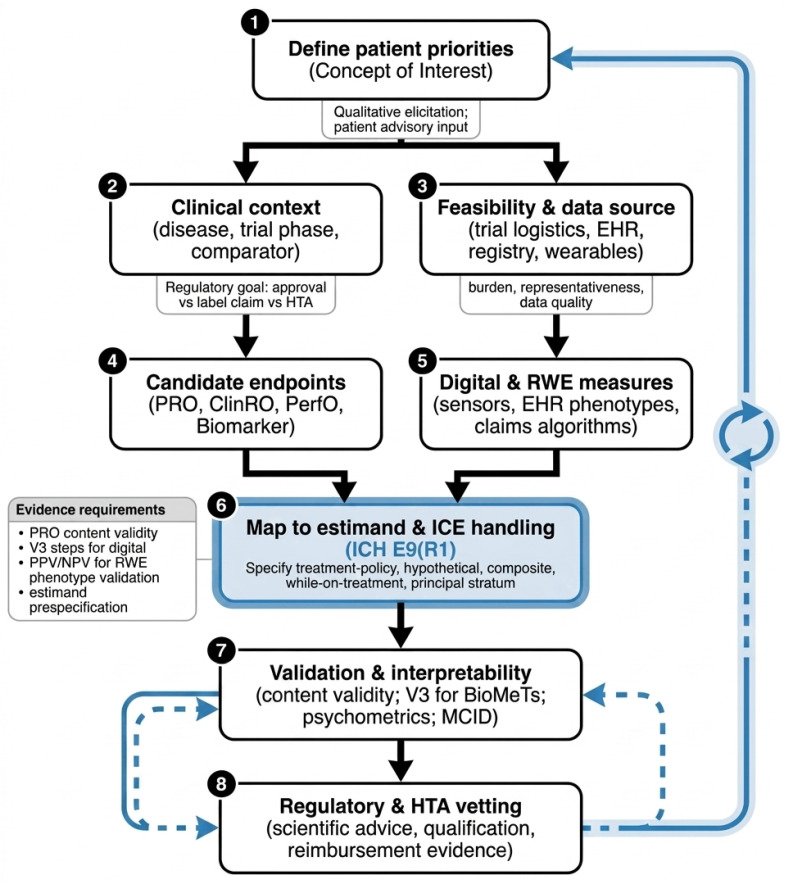
Endpoint selection flowchart—from concept to regulatory vetting. This figure illustrates an iterative lifecycle for developing patient-centric clinical outcome assessments. The framework begins with defining patient priorities (Concept of Interest) and progresses through feasibility assessments to endpoint selection (such as Patient-Reported Outcomes or digital/RWE measures). A central highlighted step (6) emphasizes the critical need to map candidate endpoints to the ICH E9(R1) estimand framework and Intercurrent Event (ICE) handling before proceeding to validation and regulatory vetting. Extensive blue arrows and the iterative icon demonstrate that this is not a linear process, requiring essential feedback loops for refinement and validation.

For digital health technologies (DHTs), the evidentiary burden is higher. The V3 Framework mandates verification, analytical validation, and clinical validation [8,15]. Critically, the recent literature suggests a simplified V3 approach for small-to-medium enterprises (SMEs), allowing for modular validation to reduce the resource intensity that often prevents smaller sponsors from adopting digital measures [16] (Figure 1).

### 3.4. Estimands: The Causal Bridge

The ICH E9(R1) addendum serves as the connective tissue between the endpoint and the clinical conclusion. An endpoint only gains clinical meaning within the context of an estimand, which consists of five attributes: (1) treatment, (2) population, (3) variable (the endpoint), (4) intercurrent events (ICEs), and (5) population-level summary [4,12,13].

A truly Patient-Centric Estimand must decide how to handle ICEs (e.g., treatment discontinuation or rescue medication use) using one of five strategies [7,18]:

1. **Treatment-Policy:** Disregards the ICE; the endpoint is measured regardless of whether the patient stayed on treatment.
2. **Hypothetical:** Estimates what the endpoint would have been had the ICE not occurred (e.g., if rescue medication had not been available).
3. **Composite:** Incorporates the ICE into the endpoint itself (e.g., failure is defined as death *or* the need for rescue meds).
4. **While-on-Treatment:** Measures the response only up until the moment the ICE occurs.
5. **Principal Stratification:** Targets a specific stratum of patients who would not experience the ICE regardless of treatment assignment.

The choice of strategy, such as using a “While-on-Treatment” for a DHT-based activity measure, fundamentally changes how the data is captured and interpreted [37].

### 3.5. Harmonization and the Learning Health System

The ILR demands that endpoints are not siloed within a single trial. Through Endpoint Harmonization, we ensure that a PRO validated in Phase 3 is also capturable in Phase 4 registries or EHRs. Utilizing Common Data Models (e.g., OMOP [30]) and standardized templates (e.g., STaRT-RWE [12]), we create a feedback loop where real-world performance informs future endpoint selection, the hallmark of a Continuous Learning Health System [7,19,30,38].

## 4. Regulatory Evolution Toward Patient-Centric Outcomes

### 4.1. Hierarchy of Evidence: Regulatory Mandates vs. Methodological Frameworks

To maintain evidentiary clarity, it is necessary to distinguish between regulatory guidance (which establishes mandatory compliance standards), consensus methodological frameworks (which provide best-practice validation steps), and empirical precedents (which serve as proof-of-concept). While regulatory guidance like ICH E9(R1) [4,13,24,25] and FDA RWE [6] dictate the “what,” methodological frameworks like V3 [5,15,16,36] and STaRT-RWE [7] provide the “how.” The integration of these disparate evidence streams into a single roadmap allows sponsors to align technical validation with the specific “Context of Use” (CoU) required for a marketing claim.

### 4.2. The Inferential Pivot: ICH E9(R1) and Causal Design

The 2019 ICH E9(R1) addendum shifted endpoint selection from an operational task to a core component of causal study design [4,13,24,25]. Regulators now require a clear distinction between the clinical objective and the statistical analysis, defined by the population, variable, and the handling of intercurrent events (ICEs) [4,13,24,25]. To resolve common methodological ambiguities, a strict distinction must be maintained between ICEs and operational missing data:

- Intercurrent Events (ICEs): Events occurring after treatment initiation that affect the interpretation of the outcome (e.g., rescue medication use). These require an estimand strategy, such as a hypothetical strategy to estimate the outcome as if the rescue medication had not been taken [4,38].
- Missing Data: Operational failures where the measurement did not occur (e.g., device non-wear). Unlike ICEs, missing data does not change the patient’s health state and requires statistical imputation (e.g., Multiple Imputation) rather than an estimand choice [38,39].

### 4.3. Maturation of Digital Standards: Objective Selection of Precedents

Parallel to the estimand shift, standards for digital health technologies (DHTs) have coalesced around the V3 Framework (verification, analytical validation, clinical validation) [5,15,16,36]. To minimize selection bias, we utilize three objective criteria for our landmark case studies (SV95C, ADAPTABLE, and RW-Response): (1) formal regulatory qualification or acceptance, (2) availability of a public evidence dossier, and (3) role as a notable precedent pathway for a novel modality [17,32,33]. The 2023 EMA qualification of Stride Velocity 95th Centile (SV95C) serves as the primary proof-of-concept for the digital lifecycle, demonstrating that wearable-derived measures can achieve primary efficacy status when V3 rigor is maintained [14].

### 4.4. RWE Infrastructure and the Validation of Computable Phenotypes

Regulatory interest in real-world evidence (RWE) has transitioned from theoretical frameworks to operational infrastructure, exemplified by the Data Analysis and Real World Interrogation Network (DARWIN-EU) [19]. This shift underscores the necessity of computable phenotypes, algorithms used to identify clinical events (e.g., myocardial infarction or disease progression) within electronic health records (EHRs) or insurance claims databases.

Because computable phenotypes are proxies for clinical events rather than direct physician observations in a controlled trial, they must be validated as if they were diagnostic instruments. Consequently, sponsors are now expected to provide evidence of an algorithm’s diagnostic accuracy using Positive Predictive Value (PPV) and Negative Predictive Value (NPV) [30,34]:

- **PPV (Reliability):** Represents the probability that a patient identified by the algorithm as having an “event” truly experienced that event in clinical reality. A high PPV is essential to ensure that the study endpoint is not diluted by “false positives,” which would bias the treatment effect toward the null.
- **NPV (Completeness):** Represents the probability that a patient the algorithm flags as “event-free” truly did not experience the event. This metric is critical for safety monitoring to ensure that toxicities or adverse outcomes are not being systematically missed (“false negatives”).

This validation typically requires a “gold standard” comparison—such as manual clinician chart review of a random sample—to establish that the RWE-derived endpoint is sufficiently credible for regulatory or HTA reliance [35,40]. This move toward objective performance thresholds represents the “professionalization” of RWE, moving it away from descriptive observational data toward a level of rigor suitable for pivotal efficacy claims.

### 4.5. Current State: Gaps and the “Bridge Study” Template

Despite this progress, significant gaps persist, primarily regarding the lack of empirical bridge studies to map traditional trial endpoints to novel counterparts. To address this lack of specific methodology, we propose a Minimum Design Template for bridge studies based on existing best practices [25,34]:

1. **Temporal Alignment:** Simultaneous capture of reference and novel measures to ensure physiological parity.
2. **Reference Standard:** Identification of a validated anchor (e.g., a ClinRO or PRO) to establish clinical meaningfulness.
3. **Agreement Metrics:** Recommended reporting of Lin’s Concordance Correlation Coefficient (CCC) and Bland–Altman plots to characterize systematic bias and precision

By aligning these technical templates with regulatory Contexts of Use (CoUs), sponsors can navigate the transition from concept to claim with greater evidentiary certainty [5,15,16].

## 5. Methodological Alignment: Navigating the Integration of Measurement and Inference

The operational success of a patient-centric endpoint depends on a bi-directional alignment between measurement science (the “V3” technical capture) and inferential science (the “ICH E9(R1)” interpretation). This section argues that the historical failure of novel endpoints often stems from “Alignment Gaps,” where a technically valid digital measure fails because its estimand strategy does not account for real-world patient behavior.

### 5.1. Estimand-Driven Design: The Causal Link

Under the ICH E9(R1) framework, an endpoint is no longer a standalone variable; it is a component of an estimand. This alignment requires trialists to pre-specify the treatment effect of interest by explicitly addressing five attributes: population, variable (endpoint), treatment, summary measure, and, most critically, the strategy for intercurrent events (ICEs) [4,13,24,25]. Crucially, to satisfy regulatory rigor, researchers must distinguish between ICEs (which change the clinical story) and missing data (which merely obscures it):

ICE Strategy (e.g., Rescue Medication): If a patient in a pain trial takes rescue analgesics, the endpoint is affected by the ICE. A hypothetical strategy estimates the pharmacological effect without rescue, whereas a Treatment-Policy strategy accepts the confounded score as the “prescribing reality” [4,13].

Missing Data Strategy (e.g., Device Non-Wear): Unlike rescue medication, a patient forgetting to wear a sensor does not alter their underlying health state. This is an operational failure requiring statistical handling (e.g., Multiple Imputation under MAR assumptions) rather than an estimand-level decision [38,39].

### 5.2. The V3 Validation Pipeline for Digital Endpoints

For digital health technologies (DHTs), alignment follows the V3 Framework (verification, analytical validation, clinical validation) [5]. Within this framework, clinical validation should align with patient-relevant concepts identified through Patient-Focused Drug Development (PFDD) [8,9,11,21].

1. **Verification:** Bench-testing to ensure the sensor captures the physical signal (e.g., acceleration) within acceptable error margins.
2. **Analytical Validation:** Demonstrating that the algorithm correctly identifies the clinical event (e.g., identifying a step from raw accelerometer data) across diverse patient movements.
3. **Clinical Validation:** Establishing that the digital output maps to a meaningful clinical concept or a “Meaningful Aspect of Health” (MAH) identified by patients [5,30].

### 5.3. Cross-Cutting Methodological Challenges

In RWE, interpretability relies on phenotype validation. If an algorithm identifies a disease event in a database, sponsors must report PPV to ensure the event is not a false positive [19,30,34]. Without this, the evidentiary weight of the RWE is insufficient to support a marketing claim [40].

The use of multiple patient-centric measures (e.g., several PRO domains) increases the risk of false positive trial results. Methodological alignment requires a pre-specified Hierarchical Testing Strategy where the primary endpoint must achieve significance before patient-centric secondary endpoints are formally tested [1,27].

A significant tension in current research is the conflict between global standardization and local relevance. A PRO or digital measure developed in one demographic may lack content validity in another due to connectivity issues or cultural differences in symptom reporting. Multi-site Transportability Studies are required to ensure that endpoints remain valid across the global trial landscape [12,14].

## 6. Evidence Generation: Comparing and Bridging RCTs and RWE

The lifecycle model posits that Randomized Clinical Trials (RCTs) and real-world evidence (RWE) are not competing paradigms but complementary engines of a single evidentiary continuum. While RCTs remain the “engine of internal validity” through strict control of confounding, RWE serves as the “engine of external validity,” capturing long-term effectiveness in heterogeneous populations. Bridging these streams requires a technical crosswalk to ensure that the patient-centric signal is preserved as data transitions from controlled to routine settings.

### 6.1. Clinical Trials: The Engine of Internal Validity and Regulatory Proof

In the RCT setting, endpoint selection is a multidisciplinary design decision that balances scientific sensitivity with regulatory acceptability. Under the ICH E9(R1) framework, this is achieved by defining the endpoint through its interaction with patient behavior (ICEs).

Trialists must define the variable within the context of intercurrent events (ICEs). A critical methodological distinction is required to avoid the common pitfall of treating clinical events as missing data:

- **Intercurrent Event (e.g., Rescue Medication):** If a patient uses rescue medication for a symptom, the observed score is no longer “pure.” A Hypothetical strategy estimating the score had the rescue not been used is often necessary to isolate a drug’s pharmacological signal for primary efficacy claims [4,27,38].
- **Missing Data (e.g., Device Non-Wear):** Unlike rescue medication, a patient forgetting a wearable sensor does not change their underlying health status. This is an operational failure that must be addressed through statistical imputation (e.g., Multiple Imputation) to recover the obscured signal without altering the estimand [38,39].

When patient experience is central, Clinical Outcome Assessments (COAs) must meet stringent FDA/EMA documentation standards. Ecological validity in DHTs (e.g., continuous actigraphy) introduces unique ICEs, such as device non-wear during symptom flares, which requires pre-specified minimum wear-time thresholds and sensitivity analyses to ensure that missingness is not correlated with the treatment effect [5,30].

### 6.2. Real-World Evidence: The Engine of External Validity

Translating trial endpoints into routine care allows for the assessment of long-term effectiveness, but it requires a diagnostic-grade validation of the data source.

RWE relies on computable phenotypes, an algorithmic logic used to reconstruct an endpoint from EHR or claims data. To meet the proven evidentiary tier, these phenotypes must undergo formal validation against a reference standard (e.g., clinician chart review), reporting specific performance metrics:

- Positive Predictive Value (PPV): High PPV is mandatory to ensure the endpoint is not diluted by false positives, which would bias results toward the null [13,14].
- Negative Predictive Value (NPV): High NPV ensures that adverse events or treatment failures are not systematically missed, safeguarding safety conclusions [7,19].

To elevate RWE to regulatory-grade evidence, researchers should apply Target Trial Emulation principles. By explicitly defining “Time Zero” (exposure initiation) and the follow-up period to mirror an RCT, researchers can avoid immortality bias and lead-time bias, which are frequent targets of reviewer criticism in RWE submissions [31].

### 6.3. The Bridge: Harmonization and the Learning Health System

The ultimate goal of the Lifecycle Framework is to ensure that an endpoint measured in a Phase 3 trial remains meaningful and measurable in a Phase 4 registry or routine EHR.

Effective bridging requires the use of Common Data Models (CDMs), such as OMOP. By mapping trial endpoints to a CDM, sponsors can run identical endpoint algorithms across global RWE databases (e.g., through DARWIN-EU), facilitating multi-database validation and rapid evidence synthesis [19,40].

To address the “underdeveloped methodological ” state of current crosswalks noted by reviewers, sponsors must conduct Bridge Studies that empirically calibrate RWE measures against trial “gold standards” using the following template [34]:

- Temporal Alignment: Simultaneous capture of trial-adjudicated events and RWE phenotypes.
- Agreement Metrics: Mandatory reporting of Lin’s Concordance Correlation Coefficient (CCC) for agreement and Bland–Altman plots to characterize systematic bias. This provides payers and regulators with the statistical confidence to utilize RWE in value-based reimbursement and long-term safety monitoring [13,34].

## 7. Critical Gaps: Barriers to Routine Adoption of Patient-Centric Endpoints

Despite the proliferation of regulatory guidance and the conceptual appeal of patient-centricity, a significant implementation gap remains. Currently, patient-centered and RWE-compatible outcomes are frequently relegated to exploratory or secondary status. Table 2 analyzes the most consequential gaps not as isolated deficits, but as an evidentiary chain where a failure in one link (such as estimand logic) compromises the entire regulatory package.

## 8. An Integrated Lifecycle Framework for Patient-Centric Endpoint Development

**Figure 2:**
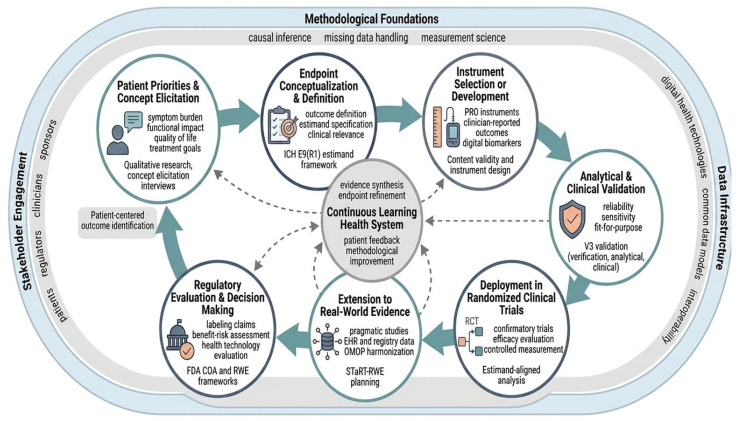
Integrated Lifecycle Framework for patient-centric endpoints development across clinical trials and real-world evidence. This schematic illustrates the iterative journey of evidence generation, moving from initial patient concept elicitation to final regulatory and HTA decision-making. The framework highlights the synergy between randomized clinical trials (RCTs) and real-world evidence (RWE), underpinned by a central “Continuous Learning Health System.” This model ensures that endpoints are not static, but are continuously refined through methodological foundations, robust data infrastructure, and active stakeholder engagement to remain both scientifically valid and clinically meaningful.

To address the structural gaps identified previously, we propose an Integrated Lifecycle Framework. This model moves beyond treating endpoints as isolated study variables, instead viewing them as dynamic assets that require continuous validation and harmonization across clinical trials and real-world care (see Figure 2).

### 8.1. Core Components of the Framework

The framework is built upon five pillars that ensure an endpoint is scientifically robust, patient-meaningful, and regulatory-ready.

1. **Concept Elicitation and Stakeholder Alignment:** The lifecycle begins with qualitative research—interviews and focus groups with patients, caregivers, and clinicians. The goal is to define the “Meaningful Aspects of Health” (MAH) and ensure the “Concept of Interest” (COI) is not lost in translation. This stage must document content validity and establish the initial “Context of Use” (CoU) before any quantitative work begins [4,11,12].
2. **Estimand Co-Specification:** Early in the design phase, clinical scientists and statisticians must co-specify the estimand per ICH E9(R1). This includes a mandatory technical distinction between intercurrent events (ICEs), which require a “Hypothetical” or “Treatment-Policy” strategy, and operational missing data, which requires a statistical imputation strategy [4,38,39].
3. **Parallel Validation Streams:** Depending on the data modality, the framework triggers specific validation pipelines:
  ○ **PROs/PerfOs:** Traditional psychometric validation (reliability, Construct Validity, MCID derivation) [10,12,21,23].
  ○ **Digital (DHTs):** The **V3 Pipeline** (verification, analytical, and clinical validation) [5,30].
  ○ **RWE: Computable phenotype validation** (PPV/NPV reporting and mapping to the OMOP Common Data Model) against a gold standard [13,14].
4. **The “Bridge” Phase:** This critical step involves empirical crosswalk studies. By analyzing datasets where patients have both adjudicated trial endpoints and real-world data capture (e.g., EHR-linked pragmatic trials), sponsors can quantify the concordance between settings. Calibration must be reported using Lin’s Concordance Correlation Coefficient (CCC) and Bland–Altman plots to characterize systematic bias [34].
5. **Iterative Regulatory Engagement:** Rather than a single submission, the framework encourages using Scientific Advice and qualification pathways (e.g., EMA’s Qualification of Novel Methodologies) to vet the evidence package iteratively. Transparency is maintained through the use of STaRT-RWE templates for RWE components [9,13] (Figure 2).

### 8.2. Operational Readiness: A Checklist for Sponsors and CROs

To transition from theory to practice, we provide a structured checklist (Table 3) to assess Endpoint Readiness for pivotal use.

## 9. Empirical Case Studies: Grounding Theory in Regulatory Reality

The transition to a patient-centric endpoint lifecycle is best illustrated by recent landmark cases where sponsors successfully navigated the complexities of estimand alignment, digital validation, and RWE integration. Rather than serving as purely illustrative examples, these cases provide insight into the evidentiary expectations, methodological trade-offs, and residual uncertainties that characterize regulatory decision-making (see Table 4).

### 9.1. Digital Qualification: SV95C in Duchenne Muscular Dystrophy (DMD)

The EMA qualification of Stride Velocity 95th Centile (SV95C) represents the definitive application of the V3 Framework. For years, DMD trials relied on the 6-Minute Walk Test (6MWT), which is prone to rater variability and clinic-day performance anxiety.

The SV95C endpoint was supported by a staged evidence package including technical verification (sensor performance), analytical validation (algorithmic accuracy), and clinical validation (association with functional outcomes). This structured approach enabled regulatory consideration of a digital endpoint in a pivotal context.

However, important limitations remain. The generalizability of SV95C beyond controlled study settings is still being evaluated, and the dependence on specific device platforms raises questions regarding standardization and cross-study comparability. Furthermore, the relationship between sensor-derived measures and clinically meaningful outcomes continues to require careful interpretation.

This case highlights that while staged validation frameworks can facilitate regulatory acceptance, they do not eliminate the need for ongoing evidence generation and contextual validation [5,14].

### 9.2. Pragmatic Trials and EHR Integration: The ADAPTABLE Study

The **ADAPTABLE** trial (Aspirin Dosing: A Patient-Centric Trial Assessing Benefits and Long-term Effectiveness) serves as an illustrative example of pragmatic RWE integration [32].

The study leveraged electronic health record (EHR) data and patient-reported outcomes collected via digital platforms to monitor a large, geographically distributed population. It demonstrated the feasibility of using computable phenotypes for cardiovascular endpoints within a pragmatic trial framework.

Nevertheless, the approach also exposed important methodological challenges, including variability in data completeness across sites, potential misclassification of outcomes, and reliance on infrastructure-specific data models. These factors underscore the dependence of RWE-based endpoints on data quality, harmonization standards, and validation procedures.

This case illustrates that while integration of EHR-derived data into clinical trials is feasible, its reliability is contingent on robust data curation and pre-specified validation strategies.

### 9.3. Bridging Oncology Outcomes: Friends of Cancer Research RW-Response

In oncology, real-world endpoints such as real-world progression-free survival (rwPFS) have historically been viewed with caution due to inconsistencies in imaging schedules and documentation practices compared to controlled trial settings.

The Friends of Cancer Research RW-Response pilot explored the use of standardized computable phenotypes and partial manual validation to align rwPFS with trial-based progression-free survival metrics. This work represents an important step toward operationalizing RWE endpoints in oncology.

However, variability in clinical documentation, heterogeneity in imaging practices, and reliance on algorithmic inference remain significant limitations. The need for manual chart review in validation highlights the ongoing tension between scalability and accuracy in RWE endpoint development.

This case underscores that phenotype validation metrics, such as Positive Predictive Value, are central to establishing credibility, but also that these metrics are context-dependent and may not fully resolve underlying data heterogeneity [33].

### 9.4. Synthesis: Moving from “Exploratory” to “Pivotal”

Taken together, these case studies suggest that regulatory acceptance of patient-centric endpoints is not driven by technological innovation alone, but by the strength and coherence of the underlying methodological framework. A consistent pattern across these examples is the requirement for a transparent and multi-dimensional evidence package addressing three core dimensions:

1. Relevance: The extent to which the endpoint reflects a meaningful aspect of patient health or experience.
2. Measurement validity: The degree to which the endpoint is reliably and accurately captured, including algorithmic performance where applicable.
3. Inferential alignment: The clarity with which the endpoint is linked to the estimand, including the handling of intercurrent events.

Importantly, these dimensions are interdependent, and weaknesses in one domain may undermine the overall acceptability of the endpoint. While recent advances demonstrate that patient-centric endpoints can be incorporated into regulatory decision-making, their transition from exploratory use to pivotal evidence remains conditional on sustained methodological rigor and context-specific validation.

## 10. Discussion

The shift toward patient-centric endpoint science represents a fundamental maturation of clinical research. Our review highlights that technical feasibility is no longer the primary bottleneck. Instead, the challenge lies in the rigorous integration of measurement, causal inference, and real-world generalizability.

### 10.1. Retrospectivity and the Need for Pre-Specification

A recurring theme across all data modalities—whether PROs, digital measures, or RWE—is the danger of retrospective endpoint definition.

- **The Risk**: Selecting or refining endpoints after data collection (e.g., choosing a specific wearable metric because it showed a signal) undermines the statistical integrity of the trial and leads to regulatory rejection.
- **The Solution**: Our Integrated Lifecycle Roadmap (ILR) emphasizes estimand co-specification before trial initiation. By pre-defining the variable, the population, and the handling of intercurrent events (ICEs), researchers move from a post hoc labeling exercise to a robust causal study design.

### 10.2. Transparency as a Regulatory Necessity

Transparency is increasingly important for regulatory credibility of modern endpoint science. For real-world evidence to be credible, the black box of computable phenotypes must be opened.

- **Phenotype Provenance**: Regulators now expect full documentation of data provenance, the journey from a raw electronic health record to a final endpoint variable.
- **Standardized Reporting**: The adoption of structured templates, such as **STaRT-RWE**, is no longer optional for high-impact submissions. These tools ensure that external validators can reproduce the endpoint and assess the risk of misclassification bias.

### 10.3. Bridging the “Evidence Silos”

A significant finding of this review is the current fragmentation between clinical trial endpoints and real-world outcomes.

- **The “Bridge” Requirement**: To make patient-centric endpoints routine, the field must invest in empirical crosswalk studies. This involves quantifying the concordance between a gold-standard trial-adjudicated event and its computable phenotype counterpart in a real-world database.
- **Impact**: Without these bridges, regulators remain uncertain about how to translate trial efficacy into real-world effectiveness or long-term safety.

### 10.4. Limitations and Generalizability

While the proposed ILR provides a robust roadmap, its application is subject to several constraints. First, as a narrative review, this work does not follow a formal PRISMA protocol. Although the search strategy was structured and the database scope was predefined, the final selection of literature and case studies remained subject to expert judgment, which introduces the potential for selection bias and limits full reproducibility. If exact Boolean strings, search dates, screening counts, and exclusion steps are not available in the manuscript record, this should be explicitly acknowledged.

Second, the proposed framework may be resource intensive. V3 validation for digital health technologies and the conversion of real-world data into OMOP Common Data Models may be difficult for small and medium-sized enterprises to implement at scale. Future work should explore streamlined validation models that preserve regulatory rigor while reducing operational burden.

Third, regulatory expectations remain heterogeneous across jurisdictions. Although standards such as ICH E9(R1) provide a common conceptual framework, evidentiary thresholds differ across agencies. For example, the EMA has established formal qualification pathways for digital measures such as SV95C, whereas the FDA often places greater emphasis on content validity and meaningfulness for patient-reported instruments.

Finally, the generalizability of digital and real-world endpoints remains constrained by the digital divide. Endpoints that perform well only in technologically advantaged populations may introduce measurement bias and limited transportability, leaving unresolved tension between global standardization and local relevance.

## 11. Conclusions

Patient-centric endpoints can bridge the gap between clinical relevance and regulatory rigor when they are considered within an Integrated Lifecycle Roadmap (ILR) rather than as isolated study elements. The primary contribution of this review is not the introduction of a wholly new methodology, but the integration and organization of existing frameworks across measurement science, causal inference, and real-world evidence generation. Achieving this requires a continuous evidentiary stream that begins with early patient concept elicitation and extends through parallel validation across clinical and real-world modalities. Central to this roadmap is the alignment of endpoints with the five ICH E9(R1) estimand strategies, including Hypothetical and Principal Stratification, to ensure that intercurrent events are handled with the statistical transparency required for pivotal claims.

By applying the V3 Framework for digital technologies and requiring diagnostic-grade PPV/NPV reporting for computable phenotypes, researchers can strengthen the technical and clinical credibility of these measures and support their progression from exploratory observations to potential primary efficacy endpoints. In addition, empirical crosswalks using agreement metrics such as Lin’s CCC may help assess whether findings are transportable from controlled trials to routine clinical practice.

Ultimately, the value of this roadmap lies in its capacity to support early stakeholder alignment and transparent evidence generation. While challenges such as the digital divide and resource intensity for SMEs remain unresolved, the ILR offers a structured way to organize current best practices and support regulatory decision-making while keeping the patient’s perspective central to endpoint development.

## Figures and Tables

**Table 1:** Strategic taxonomy and operational burdens of clinical endpoints.

Endpoint Type	Definition & Regulatory Role	Technical/Psychometric Requirements	Stakeholder Burden	Reference
**Clinical (Final)**	Direct measure of survival or morbidity.	High-fidelity adjudication; standardized definitions (e.g., VARC-3 for valves).	Low for patients; High for sites (adjudication).	[22]
**Surrogate**	Biomarker intended to substitute for clinical benefit.	Must show “biological plausibility” and “statistical surrogacy” via meta-analysis.	Minimal (lab-based).	[1,23]
**PRO**	Direct report of symptoms/function from the patient.	Content validity is paramount; must determine MCID (interpretability).	High (questionnaire fatigue); risk of missing data.	[8,11,21]
**ClinRO**	Assessment by a trained clinician (e.g., UPDRS in Parkinson’s).	Requires intensive rater training to minimize inter-rater variability.	Moderate (clinic time).	[8,35]
**PerfO**	Task-based assessment (e.g., cognitive tests, 6MWT).	Requires standardization of environment/equipment (test–retest reliability).	Moderate (physical/mental effort).	[28,34]
**Digital/BioMeT**	Passive/Active sensor data (wearables).	Must follow V3 Framework (verification -> Analytical -> Clinical).	Low (passive) to High (active tasks).	[5,15,16,36]
**RWE Phenotype**	Outcomes derived from EHR/Claims algorithms.	Requires validation against “gold standard” (PPV/NPV); OMOP mapping.	None (secondary use).	[7,19,30]

**Table 2:** Traceable synthesis of evidence gaps and regulatory consequences.

Gap Domain	Derivation from Literature & Technical Benchmarks	Specific Regulatory & HTA Consequence
**1. ESTIMAND–ENDPOINT DISCONNECT**	Mapping against ICH E9(R1) and estimand implementation literature [4,13,24,25]: Review of contemporary protocol design demonstrates persistent challenges in aligning intercurrent event (ICE) handling strategies (e.g., rescue medication, treatment discontinuation) with endpoint definition and interpretation.	Ambiguous Treatment Effect Interpretation: Regulators and HTA bodies may be unable to determine whether the observed treatment effect reflects the investigational intervention itself or confounding introduced by post-randomization events, potentially limiting interpretability and reducing evidentiary confidence.
**2. DIGITAL V3 PIPELINE INERTIA**	Mapping against the V3 Framework and digital endpoint qualification literature [5,14,15,16,36]: Current evidence indicates that many Digital Health Technologies (DHTs) successfully achieve verification and analytical validation but frequently lack sufficient clinical validation linking the measure to a meaningful aspect of health (MAH).	Limited Qualification Readiness: Device-derived measures often remain exploratory because patient relevance and clinical interpretability have not been sufficiently demonstrated for primary endpoint qualification.
**3. PHENOTYPE RELIABILITY & RIGOR**	Mapping against STaRT-RWE principles, DARWIN-EU infrastructure, and computable phenotype validation frameworks [6,7,19,30,31]: Evaluation of RWD-based endpoint studies demonstrates substantial variability in phenotype specification, validation reporting, and transparency of performance metrics such as Positive Predictive Value (PPV) and Negative Predictive Value (NPV).	Reduced Reproducibility and Regulatory Confidence: Inconsistent phenotype validation practices may reduce confidence in RWE-derived endpoints and limit their acceptability for pivotal regulatory or HTA decision-making.
**4. SCARCITY OF BRIDGE STUDIES**	Mapping against measurement agreement and endpoint comparability literature [21,34,35]: The literature demonstrates limited availability of bridge studies directly comparing adjudicated clinical trial endpoints with algorithm-derived RWE endpoints within the same patient population.	Cross-Platform Translation Uncertainty: Insufficient reporting of agreement metrics (e.g., Lin’s Concordance Correlation Coefficient, Bland–Altman analysis) creates uncertainty regarding whether RWE-derived outcomes represent the same clinical construct as centrally adjudicated trial endpoints.
**5. NUMERICAL THRESHOLD AMBIGUITY**	Mapping against regulatory qualification precedents and digital endpoint guidance [5,14,16]: Existing regulatory frameworks describe the need for reliability, validity, and interpretability, but rarely define universally accepted quantitative thresholds for evidentiary sufficiency (e.g., minimum clinically important difference [MCID], PPV targets, or agreement thresholds).	Increased Development and Negotiation Risk: The absence of clearly standardized quantitative benchmarks may contribute to prolonged regulatory interactions, increased evidentiary uncertainty, and higher operational risk for sponsors developing novel endpoints.

**Table 3:** Proposed operational checklist for patient-centric endpoint readiness.

Development Stage	Task & Documentation Requirement	Critical Success Factor
**I. Discovery**	Document Patient Concept Elicitation (Transcripts/MAH Mapping).	Direct patient quotes supporting the COI.
**II. Design**	Define ICH E9(R1) estimand and ICE handling strategies in Protocol.	Statistical/Clinical alignment on “Treatment-Policy” vs. “Hypothetical.”
**III. Technical Validation**	Complete V3 Staged Evidence (for Digital) OR Psychometrics (for PROs).	Demonstrated sensitivity to change and MCID rationale.
**IV. RWE Readiness**	Register Phenotype Algorithm; Report PPV/NPV against a Gold Standard.	Mapping to OMOP CDM for multi-database reproducibility.
**V. Bridging**	Define “Bridge Study” cohort to quantify Trial vs. RWD concordance.	Empirical correlation (e.g., Spearman’s rho) between trial and RWD events.
**VI. Regulatory**	Submit Briefing Book for Scientific Advice/Qualification.	Early consensus on “Fit-for-Purpose” thresholds.
**VII. Operations**	Plan Adherence Monitoring (eCOA/DHT) and Missing Data Sensitivity Analysis.	Minimized measurement bias and pre-specified MNAR handling.

**Table 4:** Exemplar case studies in modern endpoint science.

Case Study/Study Name	Endpoint Type	Key Methodological Achievement	Regulatory/Clinical Impact	Ref
**SV95C (Wearable)**	Digital/BioMeT	Completed full V3 pipeline; mapped sensor data to “Meaningful Aspect of Health.”	First digital endpoint qualified by EMA for primary efficacy in DMD.	[14]
**ADAPTABLE**	RWE/Pragmatic	Integrated EHR-derived phenotypes with direct-to-patient PRO reporting.	Demonstrated feasibility of large-scale pragmatic trials using RWD.	[32]
**RW-Response Pilot**	RWE (Oncology)	Developed and validated algorithms for rwPFS across disparate EHR sources.	Provided a standardized framework for RWE oncology endpoints.	[33]
**DARWIN-EU Pilots**	RWE (Multi-site)	Cross-border validation of clinical phenotypes using the OMOP CDM.	Established the infrastructure for regulator-led RWE in Europe.	[19,30]

## Data Availability

No new data were created or analyzed in this study.
